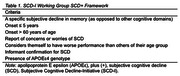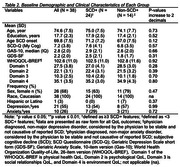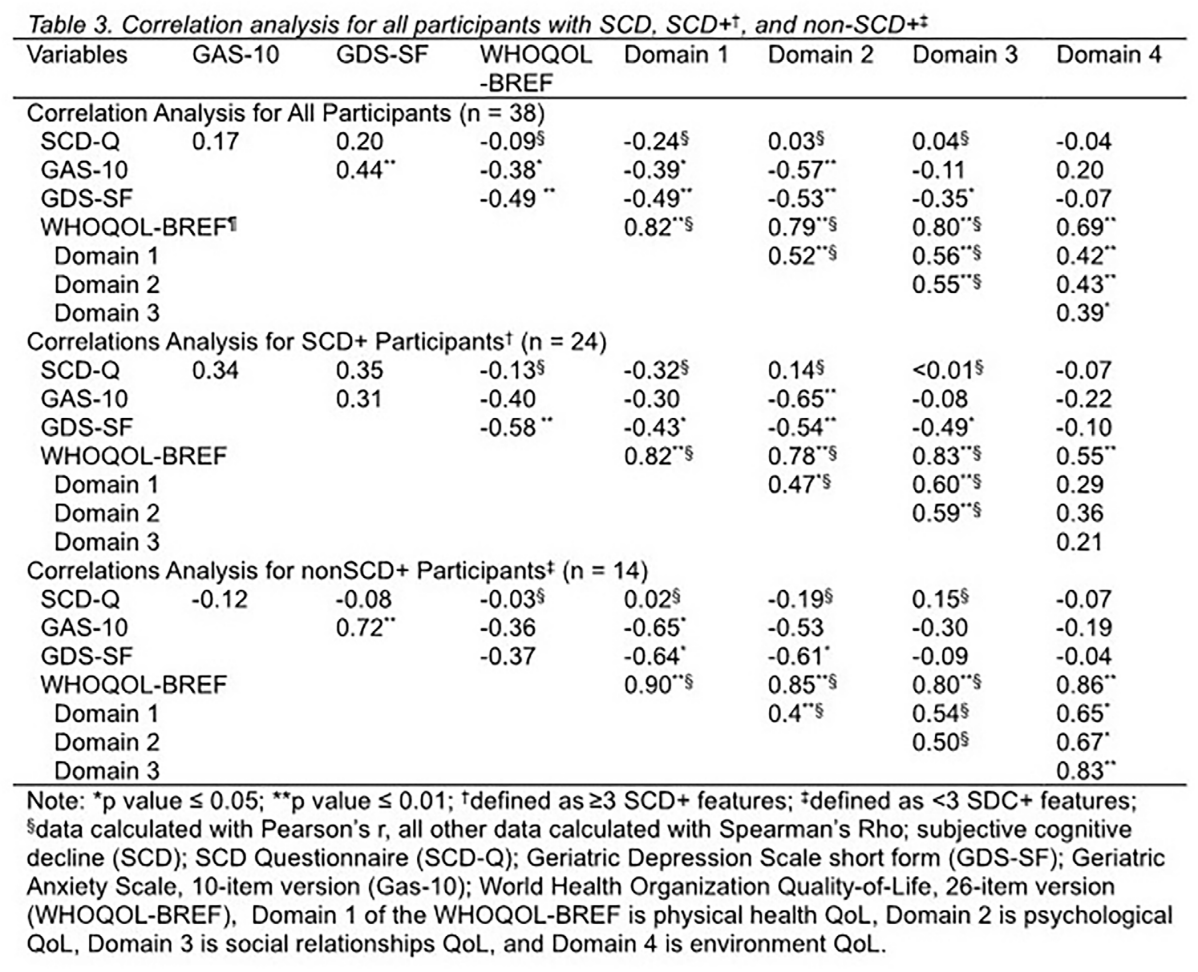# Affective symptoms and quality of life in persons experiencing subjective cognitive decline: A cross‐sectional analysis

**DOI:** 10.1002/alz.090554

**Published:** 2025-01-03

**Authors:** Rebecca Ann‐Maria Watry, Niloufar Hadidi, Mary Jo Kreitzer, Rozina Bhimani, Fang Yu, Dereck L. Salisbury

**Affiliations:** ^1^ University of Minnesota, Minneapolis, MN USA; ^2^ Arizona State University, Phoenix, AZ USA

## Abstract

**Background:**

Subjective Cognitive Decline (SCD) is a prevalent condition impacting 11.7% of older adults, which increases the risk for mild cognitive impairment and dementia. The transition to SCD and dementia is often accompanied by an increase in affective symptoms (i.e., anxiety and depression) and decreased quality of life (QoL). Affective symptoms in SCD increase the risk of Alzheimer’s disease (AD) compared to SCD alone. SCD plus (SCD+) is a condition that is known to increase the likelihood of preclinical AD, including amyloid‐beta and pathologic tau deposition. The relationships among SCD symptoms, affective symptoms, and QoL have been observed; however, they remain poorly understood. We aimed to explore the associations between SCD, affective symptoms, and QoL and identify any differences within the SCD+ subset of individuals.

**Methods:**

Community‐recruited individuals with SCD (n = 38) were divided into two groups SCD+ (those with >3 SCD+ features [Table 1]) and non‐SCD+. We used correlational analysis to determine relationships among SCD symptoms, affective symptoms, QoL overall, and four QoL domains.

**Results:**

The mean age of participants was 74.6 (7.5) years of age (Table 2). Demographic variables were not significantly different between groups. In the entire sample, significant correlations were found between anxiety and depressive symptoms (r = 0.44, *p* = <0.01); anxiety symptoms with overall (r = ‐0.38., *p* = 0.02), physical (r = ‐0.39, *p* = 0.02), and psychological QoL (r = ‐0.57, *p* <0.01); and depressive symptoms with overall (r = ‐0.49, *p* = <0.01), physical (r = ‐0.49, *p* = <0.01), psychological (r = ‐0.53, *p* = <0.01), and social (r = ‐0.35, *p* = 0.03) QoL (Table 3). Some of these correlations remained significant in the SCD+ group.

**Conclusion:**

A significantly positive correlation exists between anxiety and depressive symptoms in SCD. Multiple negative correlations exist between affective symptoms and QoL in SCD. Some of these remain significant in those with SCD+. This research provides preliminary information on the relationships between SCD symptoms, affective symptoms, and QoL. Further research with a larger sample is necessary to better understand the interactions between SCD and SCD+ with affective symptoms and QoL.